# Physical activity and sedentary behaviors (screen time and homework) among overweight or obese adolescents: a cross-sectional observational study in Yazd, Iran

**DOI:** 10.1186/s12887-021-02892-w

**Published:** 2021-09-23

**Authors:** Ali Mohammad Hadianfard, Hassan Mozaffari-Khosravi, Majid Karandish, Maryam Azhdari

**Affiliations:** 1 Department of Health Information Technology, School of Allied Medical Science, Ahvaz Jundishapur University of Medical Sciences, Ahvaz, Iran; 2 Department of Nutrition, School of Public Health, Shahid Sadoughi University of Medical Sciences, Yazd, Iran; 3 Health Research Institute, Diabetes Research Center, Ahvaz Jundishapur University of Medical Sciences, Ahvaz, Iran

**Keywords:** Overweight or obesity, Adolescents, Physical activity, Sedentary behaviors, Screen time

## Abstract

**Background:**

The growing number of adolescents who are overweight or obese (OW / OB) is a public concern. The present study was aimed to evaluate physical activity (PA) and sedentary behaviors (SB) (screen time (ST) and homework time (HT)) among Yazd OW/OB adolescents.

**Methods:**

This cross-sectional study was performed among 510 students aged 12-16 in Yazd, Iran. The general information, PA, and SB (ST and HT) were collected by interview based on the WHO standard questionnaire. Anthropometric data were assessed by precise instruments. Daily energy intake (Energy) was obtained from a 7-day food record. Nutritionist 4 software (version I) was run to estimate the energy.

**Results:**

There was a high prevalence of SB > 2h/day (97.6), ST > 2h/day (70.3%), overweight or obesity (40%), abdominal obesity (36.9%), physical inactivity (29.8%) among the students. The younger age (p = 0.014), energy (p < 0.001), no access to the yard (p < 0.001), family size ≤ 2 (p = 0.023), passive transportation, (p = 0.001), the highest school days’ HT (p = 0.033) and SB (p = 0.021), and the highest weekends’ HT among the students were the risk factors for OW/OB. The highest PA level was associated with a lower risk of OW/OB (p < 0.001). The findings were not the same in both sexes. Compared to the normal weight students, OW / OB spent more time on school days and weekdays for ST (P <0.001), HT (P <0.001, P = 0.005) and SB (P <0.001), respectively. OW/OB students showed a higher weekends’ ST (p < 0.001) and lower HT (p = 0.048) than normal-weight students.

**Conclusion:**

The prevalence of SB, ST, OW/OB, and physical inactivity were common. The school days and weekends’ HT, the school days’ SB and HT, age, energy, PA, and access to the yard, family size, and passive transportation were related to the greater chances of OW/OB students. Given that the expansion of online education and self-isolation in a new situation with COVID-19, it seems we will meet the worrying results.

## Background

Worldwide obesity among adolescents is on the rise. Obese adolescents are more likely to remain obese in adulthood [1]. The prevalence of overweight and obesity among children and adolescents aged 5-19 has risen more than 4 times from 1975 to 2016 with a similar rise among both boys and girls. According to a World Health Organization (WHO) report, the increasing prevalence of childhood obesity emanates from lifestyle changes (unhealthy dietary intake and physical inactivity patterns) in a society which leads to an energy imbalance (more calorie intake and low calories expended [2]. There is a global trend toward unhealthy dietary habits, physical inactivity, and sedentary behaviors (SB) among adolescents. However, daily energy intake was lower in the normal-weight students than the overweight or obese students [1], breakfast energy was higher in the normal-weight students [3].

Some of the most important reasons for the tendency to physical inactivity include the sedentary nature of many forms of leisure, changing modes of transportation, and increasing urbanization [1]. A study showed that more than 50% of the students (12-17 years) of the resident in Qatar reported physical inactivity and high screen time (ST) more than 2 hours (≥ 2h) on both weekdays and weekends. The prevalence of SB was higher on the weekends than weekdays. Moreover, the girl students had a more inactive pattern (physical inactivity and high ST) than the boy students as well as Qatari students than non-Qatari students. The Qatari students and non-Qataris had more ST during the weekdays and weekends, respectively [4].

Watching TV/video/DVD and doing homework for ≥ 2h, and having insufficient physical activity (PA) were related to Sri Lankan overweight adolescents aged 14-15 [5]. The prevalence of overweight or obesity and using ST ≥ 2h/day between U.S children or adolescents (6-17 years) was high (35.3% and 44%, respectively). In both genders, the high ST was related to physical inactivity. US youth with the inactive patterns (low PA along with high ST) was shown a chance of overweight at nearly two times in compared to US youth with the active patterns [6]. The prevalence of watching TV ≥ 2h (57.22 and 57.57 %), using a personal computer (PC) ≥ 2h (10.31 and 18.07%), and low PA (39.34 and 34.5%) were observed in Iranian girls and boys aged 13-18 years, respectively. Moreover, Iranian students with an unhealthy diet and overweight or obesity had a more risk for higher watching TV, using a computer, and ST. The boys were at a greater risk for excessive use of computers and ST [7]. In another study on both Iranian girls and boys (from 30 provinces of Iran, 13-18 years), the prevalence of ST (TV and PC) ≥ 2h (43.55 and 39.05%), general obesity (5.43 and 6.7%), abdominal obesity (17.58 and 19.23%), and overweight (13.9 and 14.1%) were reported, respectively [8]. The results of a systematic review in 2019 showed high ST could be one of the risk factors of overweight/obesity in children and adolescents [9]. The insufficient leisure-time PA was associated with the grade in both sexes, weekday ST, excessive use of smartphones during the weekday and weekend among the boys, studying tonight, having a work, family income, weekday ST and dissatisfaction with the own weight among the girls [10]. Polish adolescents (11-13 years) revealed that only 17% of them had the most active pattern (both low ST and high PA) with higher adherence in the boys. Physical inactivity (regardless of ST) was associated with overweight or central obesity [11].

However, the previous studies have pointed out the unfavorable impacts of physical inactivity and SB on the weight status among adolescents around the world, physical inactivity and SB were affected by socioeconomic status, gender, race/ethnicity, and geographic characteristics, and age [4, 9–12]. Therefore, it seems necessary to conduct the studies with the goal of the investigation of similar problems in each region. The present study was aimed to evaluate whether PA and SB related to the weight status among adolescents.

## Methods

### Study design and participants

The data of the present cross-sectional study were gathered among the students of Yazd, located in the center of Iran from April 20, 2019, to June 3, 2019 (before the coronavirus disease 2019 (COVID-19) pandemic).

A random multistage cluster sampling method was performed to select 569 students (12-16 years). The underweight students (N=59) were excluded from the present research based on the inclusion and exclusion criteria. Finally, 510 students were analyzed. The details for estimating the sample size were previously published [3, 13]. The eligibility criteria included the students with normal or overweight and obesity and also completing written informed consent by both students and their parents. The thin students (Body mass index (BMI) ≤ -2 Standard deviation (SD)), hospitalized within the last 6 months for any reason, used medicine such as narcotics and psychotropic, involved with the diseases such as hormonal impairment, cardiovascular disorders, malignancy were excluded from the study.

### Measurements

A general questionnaire was used to collect the information including gender, age, family size, the education levels of father or mother, access to the yard, commuting to school, and grade [7, 14]. The family size was considered based on the number of children and categorized into less and more than 2 children. The education level of the parents was categorized into 3 groups (literacy, under diploma/diploma, and college) [7]. The students were asked to determine whether they have access to the yard. Commuting to school was categorized into a) active (walking or biking) and b) passive (motor vehicles) [14]. All questionnaires were conducted by the research-trained assistants.

ST, homework time (HT), and SB were assessed for the out of the schools times [7, 8]. SB was assessed by two questions about the duration of time they spend sitting when not at the school 1) how much do you spend on HT (online/ traditional education time, reading, and studying lessons)? and 2) how much time do you spend sitting to use ST tools (TV, computers, tablets, and smartphones)?

The purpose of ST tools use was categorized into 3 groups: 1) only education, 2) leisure/ entertainment, and 3) Both of them. Leisure or entertainment was explained to the students as follows: reading or listening for a fun time, talking with friends, and recreational ST (watching TV, video, time spent in front of a computer screen, smartphone, tablet for any reason). SB was calculated by summing up ST and HT per day [5].

According to the age of the students, the PA questionnaire for the adolescents (PAQ-A) or older children (PAQ-C) was used to assess the general levels of PA for the students [15]. The score ranges for PAQ were from 1- 5. The inactive, moderate, and active students scored between 1- 1.9, 2-3.9, and 4-5, respectively [16].

Anthropometric information (weight, height, BMI, and waist circumference (WC)) were measured in the morning before completing the questionnaires and the students were lightly dressed and without shoes. BMI (kg/m^2^) was calculated by this equation: weight (kg) /height^2^ (m^2^). BMI z-score was categorized into normal students (BMI between 1SD to -2SD) and overweight or obesity (BMI ≥1SD)) [17]. Age-and-sex-specific 90^th^ percentile cut-offs of WC was used to categorize into 2 groups including normal (< 90^th^ percentile) and central/abdominal obesity (≥ 90^th^ percentile) [18].

Daily energy intake was assessed by the average of three, 24-h dietary recalls (2 weekdays and 1 weekend) for each student, individually.

### Data analysis

The analysis of quantifiable or categorical variables was conducted by means and standard deviations (SD) or frequency (number (%)). The Kolmogorov-Smirnoff test was used to test the normality distribution of data to determine the parametric or non-parametric test. The comparison of the variables was used by t-test in both genders. Binary logistic regression with ‘Enter’ method was used to model the influence of some covariates (predictors) obtained from the questionnaire on the dependent binary variable (overweight or obesity status). Adjusting was considered for the potential confounders including age, sex, grade, family size, and access to the yard based on the previous studies [7, 8, 10, 12]. SPSS statistical software package, version 26.0 (SPSS, Inc, Chicago, Illinois, USA), was applied for the statistical analyses. P < 0.05 was considered as statistically significant using 2-tailed tests.

## Results

The participants (50.6 % male and 49.4% female) were randomly selected from six high schools of Yazd. The general characterizes of the students were reported in Table 1. Unfortunately, there was a high prevalence of SB > 2h/day (97.6%), ST > 2h/day (70.3%), overweight or obesity (40%), abdominal obesity (36.9%), and physical inactivity (29.8%) among the students. The size of 44.5% of the families was reported more than four. More than half of the students (53.9%) did not have access to the yard. The main purpose of 73.8% of students who use the ST tools was leisure or entertainment. Commuting to school was passive in 54.7% of the students. It is notable that the main and significant results were shown in the text, the rest of the results were depicted in tables.

**Table 1:** Basic characterizes of the study adolescents

Variables		Frequency (%)
**Sex (N=510)**	Boy	258 (50.6)
	Girl	252 (49.4)
**Grade (N=510)**	Seventh	126 (24.7)
	Eighth	168 (32.9)
	Ninth	216 (42.6)
**Body Mass Index (BMI) (N=510)**	Normal	306 (60)
	Overweight or obesity	204 (40)
**Waist Circumstance (N=510)**	Normal	322 (63.1)
	Abdominal obesity	188 (36.9)
**Family size (N= 510)**	≤ 2	283 (55.5)
	> 2	227 (44.5)
**Education levels of mother (N=498)**	Literacy	4 (0.8)
	Under diploma/Diploma	207 (40.6)
	College	287 (56.3)
**Education levels of father (N=504)**	Literacy	6 (1.2)
	Under diploma/Diploma	156 (30.6)
	College	342 (67.1)
**Access to the Yard (N= 488)**	Yes	240 (47.1)
	No	248 (53.9)
**Commuting to the school (N=510)**	Active transportation (walking or biking)	231 (45.3)
	Passive transportation (Motor vehicles)	279 (54.7)
**Using screen time (N=504)**	Yes	499 (99)
	No	5 (1)
**The purpose of screen time use (N=503)**	Education	21 (4.2)
	Leisure/ Entertainment	371 (73.8)
	Both of them	111 (22)
**Physical activity**	Low	152 (29.8)
	Moderate	183 (35.9)
	High	175 (34.3)

Data presented by frequency (number (%))

Overweight or obese boys reported the higher times for the school days’ ST (p = 0.001), HT (p < 0.001), and SB (p < 0.001) compared to the normal-weight boys. Only ST was shown a significant increase among the overweight or obese boys on the weekends (p = 0.034). Moreover, overweight or obese boys reported a high on all week’s ST (p < 0.001), HT (p = 0.003), and SB (p < 0.001). The PA levels were higher in normal-weight boys compared to the overweight or obese boys (p < 0.001) (Table 2).

**Table 2:** The comparison of the sedentary and physical activities between the normal weight and overweight or obesity students by gender

Variables	Boy			Girl			Total		
	Normal weight	Overweight or obesity	P-value	Normal weight	Overweight or obesity	P-value	Normal weight	Overweight or obesity	P-value
**Sedentary behaviors on school days**									
**Screen time**	119.6 ± 60.3	146.32 ± 64.4	**0.001**	123.70 ± 56.4	148.78 ± 63.44	**0.001**	121.67± 58.31	147.50 ± 63.78	**< 0.001**
**Homework time**	134.6 ± 43.26	167.26 ± 71.23	**< 0.001**	139.48 ± 47.84	154.9 ± 73.32	**0.044**	137.06 ± 45.61	161.33 ± 72.33	**< 0.001**
**Sedentary behaviors time**	254.21± 73.56	313.58 ± 73.03	**< 0.001**	263.18 ± 72.81	303.67± 76.49	**< 0.001**	258.73 ± 73.2	308.82 ± 74.7	**< 0.001**
**Sedentary behaviors on weekends**									
**Screen time**	171.91 ± 75.57	193.87± 89.59	**0.034**	169.48 ± 71.17	214.9 ± 91.75	**< 0.001**	170.68 ± 73.27	203.97 ± 91.02	**< 0.001**
**Homework time**	185.07± 77.7	174.62 ± 81.32	0.298	182.37± 73.49	165 ± 75.02	0.071	183.71 ± 75.5	170 ± 78.3	**0.048**
**Sedentary behaviors time**	356.97± 103.49	368.49 ± 114.05	0.4	351.85 ± 98.99	379.9 ± 119.53	**< 0.001**	354.39 ±101.12	373.97 ± 116.57	0.051
**Sedentary behaviors on all week**									
**Screen time**	134.55 ± 54.13	159.91± 57.85	**< 0.001**	136.78 ± 48.10	167.67 ± 54.47	**< 0.001**	135.67 ± 51.11	163.64 ± 56.25	**< 0.001**
**Homework time**	149.02± 39.21	169.37± 60.27	**0.003**	151.73 ± 43.86	157.78 ± 54.85	0.359	150.39 ± 41.57	163.8 ± 57.88	**0.005**
**Sedentary behaviors time**	283.57± 67.12	329.27± 60.8	**< 0.001**	288.51 ± 63.87	325.45 ± 63.00	**< 0.001**	286.06 ± 65.44	327.45 ± 61.75	**< 0.001**
**Physical Activity**	3.58 ± 1.32	1.81 ± 0.96	**< 0.001**	3.16 ± 1.44	1.65 ± 0.97	**< 0.001**	3.36 ± 1.4	1.73 ±0.97	**< 0.001**

Data was presented by mean ± standard deviations (SD). Statistical analysis was performed using t-test. P-value < 0.05 was considered significant

Among overweight and obese girls, the school days’ ST (p= 0.001), HT (p = 0.044), and SB (p < 0.001) were higher than normal-weight girls, significantly. The weekends’ ST and SB (p < 0.001) were shown a significant increase in the overweight and obese girls. Higher times on all week’ ST and SB and lower PA levels were found in the overweight or obese girls compared to the normal-weight girls (p < 0.001) (Table 2).

In comparison to the normal-weight students, all overweight or obese students spent more time on the school days and all week ST (p < 0.001), HT (p < 0.001 and 0.005), and SB (p < 0.001), respectively and the weekends’ ST (p < 0.001). The PA levels were more in the normal-weight students than overweight or obese students (p < 0.001) (Table 2).

It is worth that the association of the independent variables with abdominal obesity in the logistic regression model was performed and their results were similar to overweight or obesity. Therefore, the relevant data on abdominal obesity was not shown in the present paper. The results of crude analyses were shown only in Tables 3, 4, and 5.

**Table 3:** Association of independent variables with overweight or obesity in the logistic regression model

Variables	Crude OR (95%CI)	P-value	Adjusted OR (95% CI)*	P-value
**Age (year)**	0.98 (0.77 , 1.237)	0.809	0.59 (0.39, 0.9)	**0.014**
**Sex (male)**				
Female	0.93 (0.647 , 1.307)	0.611	1.52 (0.76, 3.02)	0.234
**Daily energy intake**	1.003 (1.002 , 1.003)	**< 0.001**	1.003 (1.002, 1.004)	**< 0.001**
**Family Size (≤ 2)**				
**2 <**	0.32 (0.21 , 0.49)	**< 0.001**	0.48 (0.26, 0.9)	**0.023**
**Access to the Yard (No)**				
Yes	0.21 (0.14 , 0.31)	**< 0.001**	0.2 (0.1, 0.38)	**< 0.001**
**Commuting to school by (passive transportation)▀**				
Active transportation^▼^	0.241 (0.16, 0.36)	**< 0.001**	0.34 (0.19, 0.64)	**0.001**
**Schooldays. Screen time quartile (1)**				
**2**	0.9 (0.56, 1.42)	0.642	0.21 (0.06, 0.8)	**0.022**
**3**	6.5 (3.54, 11.93)	**< 0.001**	2.23 (0.34, 14.44)	0.4
**4**	2.24 (1.31, 3.82)	**0.003**	0.71 (0.08, 6.77)	0.77
**Schooldays Homework time quartile (1)**				
**3**	0.38 (0.24 , 0.60)	**< 0.001**	0.61 (0.23, 1.63)	0.323
**4**	5.25 (3.06 , 9.00)	**< 0.001**	7.11 (1.17, 43.28)	**0.033**
**Schooldays Sedentary behaviors quartile (1)**				
**2**	1.63 (0.87 , 3.04)	0.128	3.23 (1.03, 10.11)	**0.044**
**3**	2.03 (1.29 , 3.21)	**0.002**	2.38 (0.6, 9.4)	0.215
**4**	7.81 (4.56 , 13.37)	**< 0.001**	10.13 (1.42, 72.28)	**0.021**
**Weekends. Screen time quartile (1)**				
**2**	1.18 (0.72, 1.91)	0.511	1.98 (0.75, 5.2)	0.17
**3**	1.54 (0.92, 2.58)	0.101	1.48 (0.43, 5.15)	0.533
**4**	2.99 (1.76, 5.07)	**< 0.001**	3.28 (0.62, 17.24)	0.16
**Weekends. Homework time quartile (1)**				
**2**	0.73 (0.47, 1.15)	0.182	1.52 (0.55, 4.16)	0.418
**3**	1.06 (0.65, 1.74)	0.810	3.82 (1.12,13.01)	**0.032**
**4**	0.63 (0.36, 1.09)	0.096	5.55 (1.04,29.47)	**0.044**
**Weekends. Sedentary behaviors quartile (1)**				
**2**	0.99 (0.60, 1.64)	0.981	0.69 (0.24, 1.98)	0.493
**3**	0.84 (0.50, 1.41)	0.510	0.42 (0.1,1.76)	0.247
**4**	1.2 (0.72, 1.99)	0.491	0.27 (0.04,1.85)	0.184
**All week. Screen time quartile (1)**				
**2**	1.17 (0.69, 1.98)	0.551	1.8 (0.53, 6.04)	0.344
**3**	2.26 (1.34, 3.81)	**0.002**	2.6 (0.43, 15.9)	0.3
**4**	3.11 (1.83, 5.29)	**< 0.001**	1.59 (0.144, 17.32)	0.708
**All week. Homework time quartile (1)**				
**2**	0.8 (0.49, 1.31)	0.369	1.1 (0.42, 2.89)	0.841
**3**	0.57 (0.34, 0.96)	**0.034**	0.77 (0.21, 2.82)	0.689
**4**	1.79 (1.09, 2.94)	**0.021**	0.44 (0.06, 3.27)	0.418
**All week. Sedentary behaviors quartile (1)**				
**2**	1.57 (0.92, 2.7)	0.099	1.76 (0.59,5.18)	0.308
**3**	2.78 (1.62, 4.77)	**< 0.001**	0.83 (0.16, 4.35)	0.824
**4**	5.14 (2.99, 8.83)	**< 0.001**	0.52 (0.05, 4.93)	0.567
**All week. Physical activity (low)**				
**Moderate**	0.485(0.311, 0.757)	**0.001**	0.426 (0.207, 0.874)	**0.020**
**High**	0.033(0.016, 0.066)	**< 0.001**	0.039(0.013, 0.118)	**< 0.001**

*OR* Odds ratio, *CI* Confidence Interval. *P*-value < 0.05 was considered significant

^*^Adjusted for age, sex, grade, family size, access to the yard

**▀** Commuting to school by walking or biking was considered as active transportation

**▼**Commuting to school by motor vehicles was considered passive transportation

**Table 4:** Association of independent variables with overweight or obesity in the logistic regression model in boys

Variables	Crude OR (95%CI)	P-value	Adjusted OR (95% CI)*	P-value
**Age (year)**	0.837 ( 0.6, 1.167)	0.294	0.401 (0.188, 0.858)	**0.018**
**Daily energy intake**	1.002 (1.001, 1.003)	**< 0.001**	1.003 (1.002,1.004)	< 0.001
**Family Size (≤ 2)**				
**2 <**	0.462 (0.262, 0.814)	**0.008**	4.419 (1.254, 15.575)	**0.021**
**Access to the Yard (No)**				
Yes	0.185 (0.103, 0.332)	**< 0.001**	0.248 (0.075, 0.822)	**0.023**
**Commuting to school by (passive transportation)▀**				
Active transportation^▼^	0.244 (0.143, 0.416)	**< 0.001**	1.083 (0.367, 3.195)	0.885
**Schooldays. Screen time quartile (1)**				
**2**	0.892 (0.468, 1.7)	0.728	0.174 (0.023, 1.303)	0.089
**3**	5.793 (2.483,13.517)	**< 0.001**	3.430 (0.181, 64.908)	0.411
**4**	2.069 (1.008, 4.245)	**0.047**	0.507 (0.015, 17.063)	0.705
**Schooldays. Homework time quartile (1)**				
**3**	0.378 (0.197, 0.727)	**0.004**	0.2 (0.035, 1.144)	0.070
**4**	7.389 (3.302, 6.535)	**< 0.001**	1.5 (0.104, 21.562)	0.765
**Schooldays. Sedentary behaviors quartile (1)**				
**2**	1.867 (0.808, 4.31)	0.144	11.303 (1.738, 73.504)	**0.011**
**3**	2.333 (1.205, 4.519)	**0.012**	1.76 (0.189, 16.432)	0.620
**4**	10.37 (4.767, 22.561)	**< 0.001**	2.701 (0.091, 79.897)	0.565
**Weekends. Screen time quartile (1)**				
**2**	1.192 (0.62, 2.294)	0.598	2.729 (0.491, 15.168)	0.251
**3**	1.382 (0.668, 2.86)	0.383	0.799 (0.103, 6.218)	0.830
**4**	2.12 (1.025, 4.386)	**0.043**	2.316 (0.136, 39.448)	0.562
**Weekends. Homework time quartile (1)**				
**2**	0.707 (0.369, 1.354)	0.295	0.93 (0.187, 4.616)	0.929
**3**	1.161 (0.582, 2.316)	0.672	1.559 (0.232,10.502)	0.648
**4**	0.682 (0.331, 1.404)	0.299	1.215 (0.096,15.325)	0.881
**Weekends. Sedentary behaviors quartile (1)**				
**2**	0.963 (0.467, 1.984)	0.919	0.481 (0.097, 2.396)	0.372
**3**	0.869 (0.440, 1.718)	0.687	0.209 (0.02, 2.232)	0.195
**4**	0.626 (0.301, 1.301)	0.21	0.153 (0.008, 2.981)	0.215
**All week. Screen time quartile (1)**				
**2**	0.955 (0.463, 1.969)	0.9	0.826 (0.121, 5.623)	0.845
**3**	2.026 (0.997, 4.114)	0.051	1.944 (0.094, 40.201)	0.667
**4**	2.39 (1.166, 4.899)	**0.017**	1.63 (0.028, 94.137)	0.813
**All week. Homework time quartile (1)**				
**2**	0.858 (0.424, 1.738)	0.671	2.887(0.518, 16.075)	0.226
**3**	0.473 (0.224, 1)	0.05	1.736 (0.21,14.323)	0.608
**4**	2.471 (1.238, 4.933)	**0.01**	8.549( 0.304, 240.721)	0.208
**All week. Sedentary behaviors quartile (1)**				
**2**	1.884 (0.889, 3.993)	0.098	1.057 ( 0.199,5.625)	0.948
**3**	2.476 (1.148, 5.339)	**0.021**	0.392 (0.025, 6.11)	0.504
**4**	6.19 (2.827, 13.556)	**< 0.001**	1.536 (0.03, 78.886)	0.831
**All week. Physical activity (low)**				
**Moderate**	0.359 (0.179, 0.723)	**0.004**	0.132 (0.04, 0.437)	**0.001**
**High**	0.022 (0.008, 0.061)	**< 0.001**	0.004 (0.001, 0.034)	**< 0.001**

*OR* Odds ratio, *CI* Confidence Interval. *P*-value < 0.05 was considered significant

^*^Adjusted for age, sex, grade, family size, access to the yard

**▀** Commuting to school by walking or biking was considered as active transportation

**▼** Commuting to school by motor vehicles was considered passive transportation

**Table 5:** Association of independent variables with overweight or obesity in the logistic regression model in girls

Variables	Crude OR (95%CI)	P-value	Adjusted OR (95% CI)*	P-value
**Age (year)**	1.115 (0.76, 1.635)	0.578	1.081 (0.411, 2.844)	0.874
**Daily energy intake**	1.006 (1.005, 1.008)	**< 0.001**	1.007 (1.004, 1.009)	**< 0.001**
**Family Size (≤ 2)**				
**2 <**	0.221 (0.12, 0.407)	**< 0.001**	0.703 (0.175, 2.82)	0.619
**Access to the Yard (No)**				
Yes	0.206 (0.119, 0.355)	**< 0.001**	0.227 (0.058, 0.893)	**0.034**
**Commuting to school by (passive transportation)▀**				
Active transportation^▼^	0.225 (0.123, 0.41)	**< 0.001**	0.594 (0.142, 2.482)	0.475
**Schooldays. Screen time quartile (1)**				
**2**	0.929 (0.474,1.819)	0.829	0.190 (0.016, 2.198)	0.184
**3**	7.429 (3.091, 7.855)	**< 0.001**	1.158 (0.024, 54.901)	0.941
**4**	2.476 (1.112,5.514)	**0.026**	0.578 (0.004, 85.199)	0.830
**Schooldays. Homework time quartile (1)**				
**3**	0.385 (0.2, 0.74)	**0.004**	0.5 (0.067, 3.735)	0.5
**4**	3.791 (1.815, 7.917)	**<0.001**	736.588 (1.118, 485356.584)	**0.046**
**Schooldays. Overall Sedentary behaviors times quartile (1)**				
**2**	1.375 (0.53, 3.564)	0.512	2.152 (0.155, 29.866)	0.568
**3**	1.793 (0.954, 3.372)	0.070	5.178 (0.371, 72.241)	0.221
**4**	5.867 (2.77, 12.426)	**< 0.001**	5.183 (0.113, 238.025)	0.399
**Weekends. Screen time quartile (1)**				
**2**	1.163 (0.564, 2.397)	0.682	2.215 (0.317, 15.459)	0.422
**3**	1.768 (0.842, 0.713)	0.132	4.851 (0.33, 71.214)	0.249
**4**	4.343 (1.998, 9.441)	**< 0.001**	53.256 (1.773, 1599.286)	0.022
**Weekends. homework time quartile (1)**				
**2**	0.764 (0.405,1.439)	0.404	5.176 (0.403, 66.498)	0.207
**3**	0.974 (0.479,1.978)	0.941	53.685 (2.619, 1100.448)	**0.010**
**4**	0.555 (0.239, 1.288)	0.171	110.577 (1.843, 6633.313)	**0.024**
**Weekends. Overall sedentary behaviors times quartile (1)**				
**2**	0.725 (0.353, 1.489)	0.381	0.326 (0.034, 3.119)	0.331
**3**	0.778 (0.380, 1.592)	0.492	0.072 (0.002, 2.076)	0.125
**4**	0.784 (0.389, 1.58)	0.496	0.03 (0.000, 3.099)	0.139
**All week. Screen time quartile (1)**				
**2**	1.513 (0.699, 3.272)	0.293	15.83 (1.259, 198.978)	**0.032**
**3**	2.647 (1.212,5.78)	**0.015**	36.837 (0.967, 1403.868)	0.052
**4**	4.286 (1.926, 9.537)	**< 0.001**	22.237 (0.097, 5090.267)	0.263
**All week. Homework time quartile (1)**				
**2**	0.743 (0.371, 1.486)	0.401	0.286 (0.031, 2.650)	0.271
**3**	0.679 (0.33, 1.399)	0.294	0.430 (0.024, 7.824)	0.568
**4**	1.249 (0.609, 2.561)	0.544	0.009 (0.000, 4.794)	0.141
**All week. Overall sedentary behaviors times quartile (1)**				
**2**	1.272 (0.582, 2.779)	0.547	0.602 (0.06, 6.053)	0.667
**3**	3.138 (1.462, 6.736)	**0.003**	0.146 (0.004, 5.151)	0.290
**4**	4.333 (2.046, 0.176)	**< 0.001**	0.025 (0.000, 2.061)	0.101
**All week. Physical activity (low)**				
**Moderate**	0.538 (0.292,0.989)	**0.046**	1.25 (0.276, 5.66)	0.772
**High**	0.041 (0.015, 0.112)	**< 0.001**	0.069 (0.009, 0.518)	**0.009**

*OR* Odds ratio, *CI* Confidence Interval. *P*-value < 0.05 was considered significant

^*^Adjusted for age, sex, grade, family size, access to the yard

**▀** Commuting to school by walking or biking was considered as active transportation

**▼**Commuting to school by motor vehicles was considered passive transportation

In the adjusted analyses (age, sex, grade, family size, and access to the yard effects), the association between the independent variables and overweight or obesity was re-evaluated. In the adjusted analyses, there were higher chances of overweight or obesity for all students at a younger age (p = 0.014), more daily energy (p < 0.001), the smaller family size (≤ 2) (p = 0.023), no access to the yard (p < 0.001), passive transportation (p =0.001), the quartile 4 of the school days’ HT (p = 0.033), the quartile 2 (p = 0.044) and 4 (p = 0.021) of the school days’ SB, and the quartile 3 (p = 0.032) and 4 (p = 0.044) of the weekends’ HT. Moreover, low risk of overweight or obesity was presented among the students with the moderate (p = 0.001) and high (p < 0.001) levels of PA and lower school days’ ST (p = 0.022) (Table 3).

In adjusted analyses, a greater risk of overweight or obesity was related to younger age (p = 0.018), more daily energy intake (p < 0.001), the family size less than 2 (p = 0.021), no access to the yard (p = 0.023), and the quartile 2 of SB times on the school days (p = 0.011). The moderate (p = 0.001) and high (p < 0.001) levels of PA were associated with the lower risk of overweight and obesity among the boys (Table 4).

In adjusted analyses, a greater risk of overweight or obesity was related to more daily energy intake (p < 0.001), no access to the yard (p = 0.034), the quartile 4 of the school days’ HT (p = 0.046), the quartile 3 (p = 0.01) and 4 (p = 0.024) of the weekends’ HT, the quartile 2 of all week ST (p = 0.032). The highest levels of PA were associated with a low risk of overweight and obesity in the girls (p = 0.009) (Table 5).

## Discussion

The present study evaluated the association of SB, PA, and weight status among the Yazd students aged 12-16 years before the COVID-19. The current findings showed the high prevalence of SB, ST, overweight or obesity, abdominal obesity, and physical inactivity among the Yazd students. Moreover, the higher chance of overweight or obesity among Yazd students was related to the younger age, high energy intake, smaller family size, no access to the yard, passive transportation, higher school days’ HT and SB, and the weekends’ HT, and lack of moderate and high PA levels. A lower school days’ ST was inversely related to overweight or obesity. The higher chances of overweight or obesity were related to the younger age, high energy intake, smaller family size, no access to the yard, the quartile 2 of the school days’ SB, and lack of moderate and high PA levels among boys. In the girl students, there was a higher risk of overweight or obesity for high energy intake, no access to the yard, spending more time on both the weekends’ and school days’ HT, the quartile 2 of the week’s ST and lack of the high PA levels.

The present findings showed a higher prevalence of overweight and obesity [14] and abdominal obesity [8] rather than the results of the previous studies among Yazd and Iranian students. In line with the present results, some previous studies illustrated that higher PA reverses the trend of overweight [11] or obesity [19]

However, physical inactivity ≥ 2 times was reported in the girl than boy Iranian students aged 13-18 y, the analysis of total students were near the results of the present study [8]. In agreement with our results, however, some previous researches showed a relation positive was found between BMI with physically inactive, using PC ≥ 2 h/day watching TV and Video/DVD, Homework ≥ 2 h/day, and/ or high-calorie foods among adolescents of different countries [5, 8, 9, 12], there was not any data for the school days or weekends. The opposite of the current study, a positive association was presented between watching TV or using a PC ≥ 2 hours/day and PA among Iranian children and adolescents [8].

Among Irish students aged 8-11 y, a higher risk of overweight or obesity was linked to physical inactivity and SB on all week, weekdays, and weekends [20]. Our results showed a higher chance of overweight or obesity among the students is relevant to more time on the school days’ SB, HT, and ST and weekends’ HT.

Mozafarian et al. [7] illustrated that there was a negative association between the children number and spending time for SB. The study conducted by Gholami et al. [14] and the present study presented that the Yazd students who used motor vehicles had a higher BMI. A more high prevalence (64.5%) of active transportation was reported by Gholami et al. [14]. It may emanate from that the choice of high quality and facilities school has higher importance rather than near distance for the parents [14]. Attention to active transport is so important due to its role in expending a large amount of energy [14].

As the previous studies and the present study emphasized, the risk factors of overweight or obesity, were varied based on age, race, country, SES, parents’ education levels, cultural reasons and gender [11, 21]. For example, some families or countries may not encourage girls to take part in physical activities due to cultural reasons [21] or unhealthier dietary habits in boys [22] or girls [21].

The limitations of our study were a) using self-reported data on dietary intake, ST, SB and PA (however, the present questionnaire was used in previous studies [7, 8, 11, 15, 23]), b) the content of dietary intake were not assessed for the current study, the interpretation should be done with caution given that this is a cross-sectional study, c) the consideration of more confounder factors including the quality and quantity of dietary intake, sleep habits, socioeconomic status (SES), parent history of overweight or obesity, and etc., d) the lack of the data for all year (the data was collected at a particular point of time (only for an academic year), the adolescents’ distance from school was not considered, and finally SB was assessed for the activities out of the school time.

Our study had some strong aspects including 1) sample size was nearly large; 2) data were collected from both gender; 3) the analysis was performed based on both genders as well as the weekdays and weekends apart from all week; 4) the evaluation of the relationship between overweight or obesity with daily energy intake, PA and the times spent on ST, HT, and SB; 5) the analysis for abdominal obesity was performed (due to the similarity to the present results did not show); 6) the presence of the qualified and same assessors while filling in the questionnaire allowed adolescents to clarify uncertainties and reduce the biases; and finally, the data for ST and SB was comprehensive.

According to the current and previous findings [3–7, 11, 13, 15], it seems to be necessary that policymakers should take the measures to modify lifestyles to reduce the prevalence of overweight or obesity during adolescence.

However, the socioeconomic issues affected on social isolation, negatively or positively, a reduction in several health-related behaviors (insufficient PA, increased sleep time, reduced fruit and vegetable consumption, too much ST) was shown during COVID-19 lockdown [24, 25].

We suggest performing a large and comprehensive study to collecting the data for the evaluation and comparison of PA and SB between the academic year and summer holidays as well as during and before the COVID-19 lockdown. Given that the changes in lifestyle during the COVID-19 confinement (mandatory online education, the closure of the school, higher sleep time, and the more accessible cyberspace) may worsen health-related behaviors [24, 25] . In addition, it seems to be necessary to conduct more researches on more risk factors of obesity in the different regions of the world given that the risk factors of overweight or obesity were too wide [11].

## Conclusion

The prevalence of SB, ST, overweight or obesity, abdominal obesity, and physical inactivity was common among Yazd students. Among the students, the daily energy intake, PA, younger age and family size, passive transportation, access to the yard, school days’ HT and SB and the weekends’ HT were related to overweight or obesity. As the results were presented, the chances of overweight or obesity were dissimilar in boys and girls. Given that the high prevalence of unhealthy lifestyles among adolescents during regular life situations, it will be a major alarm for the decision-makers on the adolescents’ health especially during and after the COVID-19 pandemic.

## Data Availability

The datasets used and/or analyzed during the current study are available from the corresponding author on reasonable request.

## References

[1] Noncommunicable Diseases: Childhood Overweight and Obesity. Available online:B https://www.who.int/news-room/q-a-detail/noncommunicable-diseases-childhood-overweight-and-obesity. Accessed 19 Oct 2020.

[2] Obesity and Overweight. Available online: https://www.who.int/news-room/fact-sheets/detail/obesity-and-overweight. Accessed 1 Mar 2021.

[3] Karandish M, Mozaffari-Khosravi H, Hadianfard AM, Azhdari M, Amiri R, Mirzavandi F (2020). Distribution of Nutrients in Breakfast and Midmorning Snacks among Overweight or Obese Adolescents of Yazd, Iran. J Nutr Food Security.

[4] Al-Thani M, Al-Thani A, Alyafei S, Al-Kuwari M, Al-Chetachi W, Khalifa S (2018). Prevalence of physical activity and sedentary-related behaviors among adolescents: data from the Qatar National School Survey. Public Health.

[5] Godakanda I, Abeysena C, Lokubalasooriya A (2018). Sedentary behavior during leisure time, physical activity and dietary habits as risk factors of overweight among school children aged 14–15 years: case control study. BMC Res Notes.

[6] Sisson SB, Broyles ST, Baker BL, Katzmarzyk PT (2010). Screen time, physical activity, and overweight in US youth: National Survey of Children's Health 2003. J Adolesc Health.

[7] Mozafarian N, Motlagh ME, Heshmat R, Karimi S, Mansourian M, Mohebpour F, et al. Factors associated with screen time in Iranian children and adolescents: the CASPIAN-IV study. Int J Prev Med. 2017;8:31. 10.4103/ijpvm.IJPVM_36_17 PMC5439292 28567233

[8] Kelishadi R, Qorbani M, Djalalinia S, Sheidaei A, Rezaei F, Arefirad T (2017). Physical inactivity and associated factors in Iranian children and adolescents: the Weight Disorders Survey of the CASPIAN-IV study. J Cardiovasc Thoracic Res.

[9] Fang K, Mu M, Liu K, He Y (2019). Screen time and childhood overweight/obesity: A systematic review and meta-analysis. Child Care Health Dev.

[10] Lourenço CL, da Silva VD, Mendes EL (2020). Prevalence and associated factors with insufficient leisure-time physical activity of adolescents: results of a cross-sectional school population-based study. Revista sobre la infancia y la adolescencia.

[11] Górnicka M, Hamulka J, Wadolowska L, Kowalkowska J, Kostyra E, Tomaszewska M (2020). Activity–Inactivity Patterns, Screen Time, and Physical Activity: The Association with Overweight, Central Obesity and Muscle Strength in Polish Teenagers. Report from the ABC of Healthy Eating Study. Int J Environ Res Public Health.

[12] Omorou AY, Manneville F, Langlois J, Legrand K, Böhme P, Muller L (2020). Physical activity rather than sedentary behaviour is socially determined in French adolescents with overweight and obesity. Prev Med.

[13] Mozaffari-Khosravi H, Karandish M, Hadianfard AM, Azhdari M, Sheikhi L, Tabatabaie M, et al. The relationship between sleep quality and breakfast, mid-morning snack, and dinner and physical activity habits among adolescents: a cross-sectional study in Yazd, Iran. Sleep Biol Rhythms. 2021:19(1):79–84.

[14] Gholami S, Rahmanian M, Jam Ashkezari S, Hazar N, Aghaee-Meybody SMR, Namiranian N (2019). The Prevalence of Obesity and Overweight and Its Relevance to Transportation Among Primary School Students: Yazd, Iran; 2015. Int J School Health.

[15] Kowalski KC, Crocker PR, Donen RM (2004). The physical activity questionnaire for older children (PAQ-C) and adolescents (PAQ-A) manual. College of Kinesiology, University of Saskatchewan.

[16] Adeniyi AF, Okafor NC, Adeniyi CY (2011). Depression and physical activity in a sample of nigerian adolescents: levels, relationships and predictors. Child Adolesc Psychiatry Ment Health.

[17] Md O, Onyango AW, Borghi E, Siyam A, Nishida C, Siekmann J (2007). Development of a WHO growth reference for school-aged children and adolescents. Bull World Health Organ.

[18] Xi B, Zong X, Kelishadi R, Litwin M, Hong YM, Poh BK (2020). International waist circumference percentile cutoffs for central obesity in children and adolescents aged 6 to 18 years. J Clin Endocrinol Metab.

[19] Amiri P, Naseri P, Vahedi-Notash G, Jalali-Farahani S, Mehrabi Y, Hamzavi-Zarghani N (2020). Trends of low physical activity among Iranian adolescents across urban and rural areas during 2006–2011. Sci Rep.

[20] Keane E, Li X, Harrington JM, Fitzgerald AP, Perry IJ, Kearney PM (2017). Physical activity, sedentary behavior and the risk of overweight and obesity in school-aged children. Pediatr Exerc Sci.

[21] Allafi A, Al-Haifi AR, Al-Fayez MA, Al-Athari BI, Al-Ajmi FA, Al-Hazzaa HM (2014). Physical activity, sedentary behaviours and dietary habits among Kuwaiti adolescents: gender differences. Public Health Nutr.

[22] Zhang J, Yi Z, Feng XQ, Li WR, Lyu YB, Astell-Burt T (2018). Gender differences in the prevalence of overweight and obesity, associated behaviors, and weight-related perceptions in a national survey of primary school children in China. Biomed Environ Sci.

[23] Kelishadi R, Motlagh ME, Bahreynian M, Gharavi MJ, Kabir K, Ardalan G, et al. Methodology and early findings of the assessment of determinants of weight disorders among Iranian children and adolescents: The childhood and adolescence surveillance and prevention of adult Noncommunicable Disease-IV study. Int J Prev Med. 2015;6:77. 10.4103/2008-7802.162953 PMC4564901 26425332

[24] López-Bueno R, López-Sánchez GF, Casajús JA, Calatayud J, Tully MA, Smith L (2021). Potential health-related behaviors for pre-school and school-aged children during COVID-19 lockdown: A narrative review. Prev Med.

[25] López-Bueno R, López-Sánchez GF, Casajús JA, Calatayud J, Gil-Salmerón A, Grabovac I, et al. Health-related behaviors among school-aged children and adolescents during the Spanish Covid-19 confinement. Front Pediatr. 2020;8:573. 10.3389/fped.2020.00573 PMC7516648 33042917

